# Auditory processing in individuals with auditory neuropathy

**DOI:** 10.1186/1744-9081-1-21

**Published:** 2005-12-01

**Authors:** Ajith U Kumar, M Jayaram

**Affiliations:** 1 Junior Research Fellow, Department of Audiology, All India Institute of Speech and Hearing, Manasagangothri, Mysore, Karnataka, 570006, India; 2 Director, All India Institute of Speech and Hearing, Manasagangothri, Mysore, Karnataka, 570006, India

**Keywords:** Auditory neuropathy, speech perception, temporal processing, LLRs and MMN

## Abstract

**Background:**

Auditory neuropathy is a disorder characterized by no or severely impaired auditory brainstem responses in presence of normal otoacoustic emissions and/or cochlear microphonics. Speech perception abilities in these individuals are disproportionate to their hearing sensitivity and reported to be dependent on cortical evoked potentials and temporal processing abilities. The disproportionate loss of auditory percept in presence of normal cochlear function is suggestive of impairment of auditory neural synchrony.

**Methods:**

We studied the auditory evoked potentials and psychophysical abilities in 14 adults with auditory neuropathy to characterize their perceptual capabilities. Psychophysical tests included measurement of open set speech identification scores, just noticeable difference for transition duration of syllable /da/ and temporal modulation transfer function. Auditory evoked potentials measures were, recording of P_1_/N_1_, P_2_/N_2 _complex and mismatch negativity (MMN).

**Results:**

Results revealed a significant correlation between temporal processing deficits and speech perception abilities. In majority of individuals with auditory neuropathy P_1_/N_1_, P_2_/N_2 _complex and mismatch negativity could be elicited with normal amplitude and latency. None of the measured evoked potential parameters correlated with the speech perception scores. Many of the subjects with auditory neuropathy showed normal MMN even though they could not discriminate the stimulus contrast behaviorally.

**Conclusion:**

Conclusions drawn from the study are

1. Individuals with auditory neuropathy have severely affected temporal processing.

2. The presence of MMN may not be directly linked to presence of behavioral discrimination and to speech perception capabilities at least in adults with auditory neuropathy.

## Background

Auditory neuropathy (AN) is recently described hearing disorder characterized by abnormal auditory nerve functioning in presence of normal cochlear receptor hair cell activity [1]. The clinical findings that define auditory neuropathy are

a) Presence of outer hair cell integrity in evoked otoacoustic emission or cochlear microphonics.

b) Absence of synchronized neural activity at the level of 8^th ^nerve and brainstem.

Though the audiometric and electrophysiological findings are consistent with the 'retro outer hair cell dysfunction' exact site(s) of the pathology is yet to be determined. Some possible sites of lesion that could produce the audiometric and electrophysiological profile of AN include: inner hair cells, synaptic junction between inner hair cell and type I afferent nerve fibers, spiral ganglion cells, specific damage or demyelinization of type I auditory nerve fibers [1-3]. Therefore, AN consists of many varieties depending on the sites of lesion [4]. Speech perception ability in these patients also varies considerably. Some patients perform at the levels expected for patients with comparable degrees of sensory hearing loss and others show speech understanding which is disproportionate to their degree of hearing loss [5,6].

Speech perception abilities in these patients appear to depend on the extent of suprathreshold temporal distortions of cues rather than access to speech spectrum, unlike the patients with sensory hearing loss [7,6]. Zeng et al [8] reported the abnormal results on two measures of temporal perception in their group of children with AN: (i) gap detection threshold (identification of silence embedded in within the bursts of noise) and (ii) temporal modulation transfer function (measure of sensitivity to slow and fast amplitude fluctuation). They also found a correlation between temporal modulation transfer function (TMTF) and speech perception abilities in their patients. Rance et al [6] also reported poor performance on the task involving timing cues (TMTF, temporal aspects of frequency discrimination) in a group of 14 children with AN. These temporal processing abnormalities had significant correlation with speech perception abilities. They attributed the speech perception scores that are disproportionate to pure tone hearing loss to these suprathreshold temporal processing deficits.

Another factor that is reported to be related to speech perception abilities in these individuals is cortical evoked event related potentials. Rance et al [5] reported that a subgroup of children with AN, who had recordable cortical evoked potential performed well on open set speech perception task and derived significant benefit from amplification. In contrast, subjects who had no recordable cortical evoked potential performed poorly on the same tasks. From this observation they concluded that presence of cortical auditory evoked potential reflects some amount of preserved synchrony in central auditory system which contributes to better speech understanding despite the distortion that occurs at 8^th ^nerve and auditory brainstem in these individuals.

Speech perception process can be investigated in neurophysiological as well as psychophysical perspective. An important aspect of this study is use of a combined neurophysiological and psychophysical approach. With this multidisciplinary technique we hope to gain insight into both stimulus representation and processing in individuals with AN. This study is sought to explore the relation between their psychoacoustic abilities and evoked potential parameters, in a group of adults with auditory neuropathy. Psychophysical experiments included were measurement of open set speech identification scores, just noticeable difference (JND) for transition duration of the syllable /da/ and temporal modulation transfer function. Auditory evoked potentials measures included recording of N_1_/P_1_, N_2_/P_2 _and Mismatch negativity (MMN) potentials.

## Methods

Study was carried out in two phases, first phase involved psychophysical experiments and auditory evoked potentials were measured in the second phase.

### Subjects

Two groups of subjects participated in the study. The first group consisted of 14 individuals with AN (16 to 30 years with the mean age of 23 years) and second group consisted of age and gender matched 30 normally hearing subjects. All AN subjects were recruited from Department of Audiology, All India Institute of Speech and Hearing, Mysore. No subject complained about any middle ear disease (assessed using otoscopy, tympanometry and clinical history), noise exposure or ototoxic drug usage. Results of different audiological measurements of AN subjects are shown in Table 1. As all the subjects had symmetrical hearing loss, (symmetrical hearing loss was operationally defined as the difference in thresholds between two ears at corresponding frequencies within 15 dB), pure tone thresholds were measured again with loudspeakers and these measurements were considered for all future purpose. Furthermore, subjects in the normally hearing group had their hearing thresholds within 15 dB HL at octave frequencies between 250 Hz to 8 kHz and normal results on immittance evaluation. All the subjects were native speakers of Kannada, a South Indian Dravidian language.

**Table 1:** Audiometric and electrophysiological details of auditor neuropathy subjects.

SN	Age/sex	PTA (.5, 1 and 2 KHz)	Speech identification scores	OAE	ABR	Acoustic reflex	Efferent suppression	Configuration
1	16/F	45.00	45.00	Present	Absent	Absent	0.2	Raising
2	16/M	13.00	84.00	Present	absent	Absent	0.0	Peaked
3	30/M	23.00	80.00	Present	absent	Absent	0.4	Peaked
4	24/F	25.00	38.00	Present	absent	Absent	0.0	Peaked
5	16/M	40.00	.00	Present	absent	Absent	0.1	Raising
6	26/M	45.00	4.00	Present	absent	Absent	0.0	Raising
7	23/F	45.00	5.00	Present	absent	Absent	0.0	Raising
8	23/M	75.00	.00	Present	absent	Absent	0.1	Flat
9	27/M	20.00	50.00	Present	absent	Absent	0.0	Peaked
10	23/M	40.00	86.00	Present	absent	Absent	0.3	Peaked
11	24/M	23.00	8.00	Present	absent	Absent	0.0	Peaked
12	25/F	45.00	.00	Present	absent	Absent	0.0	Raising
13	28/M	5.00	90.00	Present	absent	Absent	0.3	Peaked
14	25/F	10.00	95.00	Present	absent	Absent	0.2	Peaked

PTA = Pure tone average

OAE = Otoacoustic emissions

ABR = Auditory brainstem responses

### Psychophysical tests

The experiment protocol consisted of speech identification score testing, measurement of JND for transition duration of /da/ and TMTF.

#### (a) Speech identification testing

Only AN subjects participated in this experiment. Vandana's speech identification test in Kannada was used to assess the open set speech perception abilities in the subjects. This test consists of 50 bisyllabic meaningful words in Kannada. Validity and reliability of this test on native speakers of Kannada have already been established by Vandana, [9]. Recorded material was presented at 'comfortable level' which ranged between 30 to 40 dB SL ref: Average thresholds at 500 Hz, 1 kHz, and 2 kHz, using MA-53 clinical audiometer through a loudspeaker kept at 1 m distance and 0° azimuth. Output of the loudspeaker was calibrated using Quest 1800 sound level meter and Quest 4180 free field microphone. A calibration tone recorded before the test material was used to adjust the Vu meter deflection to zero. The test was carried out in a quiet listening condition and each stimulus was presented in isolation without being embedded in a carrier phrase. The subjects were required to repeat each stimulus and a percentage of correct identification was determined. All the subjects were screened for misarticulations using Kannada Articulation Test [10]

#### (b) JND measurements

Both AN and normal listeners participated in this experiment. Stimulus was derived from retroflex /da/ uttered in isolation, by a 25 year old male native speaker of Kannada. The spoken was digitally recorded on a data acquisition system at 44 kHz sampling frequency. The transition duration was identified using both spectral and wave form view of the stimulus. Transition duration was lengthened up to 'original transition duration +100 ms' in 10 ms steps by means of Pitch Synchronized Overlap and Add (PSOLA) technique. PSOLA performs the lengthening of the stimulus in time domain and preserves most of physical characteristics of the stimulus such as spectral shape, amplitude distribution, and periodicity [11].

Subjects were tested individually in a sound attenuated room. Signals were played via a PC, at a sampling frequency of 44 kHz and were subsequently fed to a MA-53 audiometer. Subjects received the signals through audiometer's loudspeaker kept at a distance of 1 m and 0° azimuth. Presentation level of the stimulus was fixed at 30 dB SL ref: Average thresholds at 500 Hz, 1 kHz, and 2 kHz. Stimuli were presented at equal presentation level to compensate for the audibility in individuals with auditory neuropathy. JND was determined using an adaptive tracking technique (PEST) with AX same difference discrimination paradigm (in this A = anchor stimulus, X = Variable stimulus and subjects task is to indicate whether A is same as X or not). Inter stimulus interval between anchor and variable stimulus was 500 ms. Step size and the direction of variable stimulus were changed according to rules of PEST [12]. The subject's JND was determined by calculating the difference in transition duration between anchor and variable stimuli that is required to achieve a performance level of 69% correct responses. Test trials also included equal number of catch trials. Catch trial consisted of either two identical anchor or two identical non anchor stimuli.

#### (c) Temporal modulation transformer function

Both AN and normal listeners participated in this experiment. Modulation detection thresholds were measured by determining the sensitivity to sinusoidal amplitude modulation as a function of modulation frequency. Presentation level of the stimulus was kept at 30 dB SL ref: Average thresholds at 500 Hz, 1 kHz, and 2 kHz. Stimulus was presented through a loud speaker kept at a distance of 1 m and 0° azimuth. Stimuli were presented at equal sensation level to compensate for the audibility in patients with auditory neuropathy. A broad band noise was generated and controlled digitally to measure TMTF. Broad band noise had a duration of 500 ms and ramp of 2.5 ms. The modulated signal was derived by multiplying the 500 ms white noise by a dc shifted sine wave. The depth of the modulation was controlled by varying the amplitude of modulating sine wave. Modulation depth for the various stimuli varied between 0 to -30 dB and step size was 3 dB. Modulation detection thresholds were measured for 5 frequencies; 4 Hz, 16 Hz, 32 Hz, 64 Hz, 128 Hz, and 200 Hz,. Procedure was same as that described for the measurement of JNDs. In all the subjects at least at one modulation frequency the presentation level was changed and modulation detection threshold was rechecked to ensure that subjects are not using the loudness judgments.

### Auditory evoked potential measurements

In this experiment both normal hearing subjects and individuals with AN participated. The cortical evoked potentials were obtained in one session lasting less than 15 min. The subjects were seated in a comfortable position to ensure relaxed posture to minimize muscular artifacts. They were instructed not to pay attention to the stimuli. A silent cartoon movie was played to minimize the possibility of active attention. The stimuli was unmodified /da/ and synthesized /da/ in which transition duration was lengthened by 100 ms. This was decided on the basis of a pilot study measuring the behavioral JND in AN subjects. Synthesis technique was same as the one used for psychoacoustic testing. These two contrasts were presented in an odd ball paradigm. Stimuli were presented at 'comfortable level' to both ears (usually 30 to 40 dB SL, Ref: Average thresholds at 500 Hz, 1 kHz, and 2 kHz) through EAR-3A insert receiver. IHS smart EP module was used to control the stimulus presentation and acquisition of evoked potential. Conventional recording techniques were used. After skin preparation at electrode site, silver-chloride disc electrodes were placed at C_z_, with ipsilateral mastoid as reference, using conductive electrode paste and adhesive tape. Ground electrode was placed at F_z _Data was acquired after ensuring that the impedance at all electrode sites was within permissible limits. The protocol used for recording is shown in Table 2.

**Table 2:** Protocol used for evoked potential testing

Stimulus	Standard – unmodified /da/ Deviant – Synthesized /da/
Intensity	30 to 40 dB SL
Probability	5:1
Repetition rate	1.1/s
Analysis time	500 ms
Gain	75000
Band Pass Filter	1 to 30 Hz
Transducer	EAR-3A insert ear phones

In order to probe the representation of these two stimulus contrasts at pre-attentive neural level MMN responses were derived from recorded cortical evoked potentials. MMN is a passively elicited cortical evoked potential that is known to reflect the brain's response to an acoustic change [13]. The MMN is seen as a negative deflection around 200 ms after stimulus presentation. MMN was identified in the difference wave between frequent and infrequent recordings. Grand average waveform was also constructed by utilizing the individual waveform which had MMN.

## Results

### Psychophysical tests

#### (a) Open set speech identification test

Open set speech identification scores in individuals with AN varied considerably. The mean speech identification score was 41.7% (SD: 38.8%), but scores ranged from 0% to 95%. Speech identification scores correlated with low frequency (250 Hz, 500 Hz, 1000 Hz) hearing thresholds (r = 0.67, p = 0.001) but not with the high frequency hearing thresholds (2000 Hz, 4000 Hz and 8000 Hz r = 0.3, p = .234).

#### (b) JND measurements

**Figure 1:**
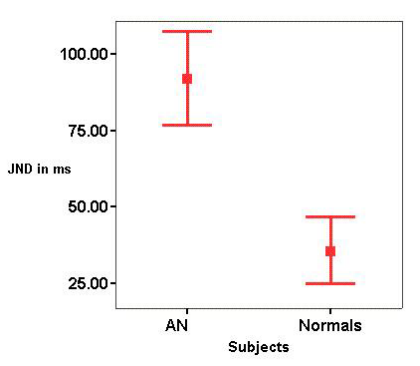
Mean and SD (error bars show 1 SD) of JND in transition duration for the auditory neuropathy (AN) group and normally hearing subjects.

Figure 1 shows the mean and SD values of JND in transition duration for stimuli /da/. Independent sample 't' test showed a significant difference between two groups at .001 level. Of 14 subjects 10 could not differentiate the stimuli that differed in transition duration by as much as 100 ms. Four subjects whose JND were less than 100 ms also had their open set speech identification scores more than 80%.

#### Temporal modulation transfer function

**Figure 2:**
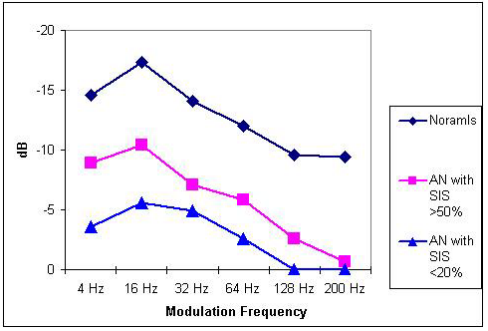
TMTF for the auditory neuropathy (AN) group and normally hearing subjects. AN20 = TMTF for auditory neuropathy subjects with speech identification scores less than 20%. AN50+ = TMTF for auditory neuropathy subjects with speech identification scores more than 50% Normal = TMTF for normally hearing subjects.

Figure 2 shows the TMTF for subjects with normal hearing and auditory neuropathy. Normal hearing listeners were most sensitive to slow temporal fluctuation and became less sensitive as the fluctuation rate was increased. Similar trend was noticed in individuals with AN. Average peak sensitivity of normal hearing listeners was -17.36 dB. In contrast, average peak sensitivity for auditory neuropathy group was -6.6 dB (SD: 5.4 dB). At higher modulation frequencies many of the AN (12 subjects) subjects did not even detect a modulation of depth of 0 dB (100%). Peak sensitivity of AN group tended to fall in two distinct categories. Eight individuals had peak sensitivity of more than -10.4 dB and 7 of these patients had open set speech identification scores more than 50%. Six subjects had peak sensitivity less than -5.6 dB and 5 of them had speech identification scores of less than 20%. One subjects in each category had paradoxical results on speech perception and TMTF results. When data from individual subjects were examined speech identification scores and temporal modulation transfer function in these two subjects were in extreme. Hence these two subjects were treated as outliers and when data from these two subjects were excluded a significant correlation was observed between peak sensitivity and speech identification scores. No relation could be established between JND measurements and TMTF.

### Auditory evoked potential measurements

**Figure 3:**
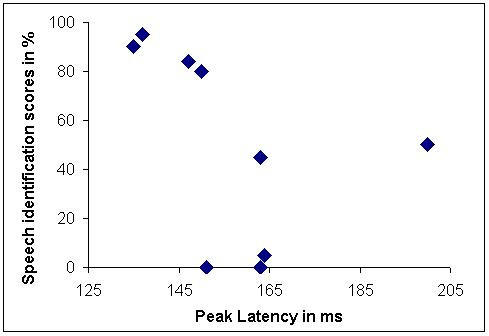
Scatter plot between speech identification scores and peak latency of MMN in auditory neuropathy subjects.

**Figure 4:**
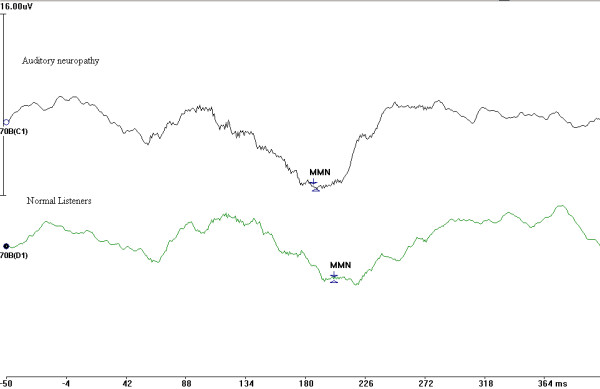
Grand averaged MMN wave form in the auditory neuropathy group and normally hearing subjects.

Before doing the analysis all the wave forms were corrected for baseline EEG activity by subtracting the pre-stimulus electrical activity (for 50 ms before the presentation of stimulus). Table 3 shows, the latencies and amplitudes of peaks P_1_, N_1_, P_2 _and N_2 _for AN and normal hearing group. P_2_/N_2 _complex was present is all 14 individuals whereas P_1_/N_1 _complex was not present in 4 subjects. Whenever LLRs were present, latency and amplitudes were within normal range. Presence or absence of LLR peaks did not bear any relation to the speech identification scores. Pearson's product moment correlation failed to evidence any significant correlation between evoked potential parameters and other psychophysical test results. Table 4 shows latency, amplitude and area of MMN parameters. Area of the MMN was determined by calculating the area between wave and baseline and took into account both the duration and amplitude of MMN response. In 5 of 14 subjects, MMN could not be elicited. Pearson's product moment correlation was performed between MMN parameters and other psychophysical measures. Only peak latency of MMN evidenced a significant correlation with speech identification scores. As the number of subjects with MMN present was less, to interpret the results of correlation, a scatter plot was drawn between MMN peak latency and speech identification scores. As seen from the scatter plot (Figure 3), no trend could be observed between MMN peak latency and speech identification scores. Figure 4 shows the grand average of MMN waveform in AN subjects and normal hearing listeners. Whenever the MMN was present in individuals with AN, wave form was indistinguishable from normal listeners.

**Table 3:** Mean and SD (values in parenthesis) of amplitude and latencies of LLR components in both groups

		P_1_	N_1_	P_2_	N_2_
AN subjects	Amplitude (in μV)	2.8 (0.9)	0.9 (0.8)	2.8 (2.08)	-1.1 (2.3)
	Latency (in ms)	81 (16.2)	125.4 (23.04)	154.1 (27.1)	205 (23)
Normal subjects	Amplitude (in μV)	2.5 (0.6)	-0.5 (0.5)	2.8 (1.5)	-1.6 (1.5)
	Latency (in ms)	69 (15.2)	120.5 (23.5)	145.3 (25.6)	200.2 (26.3)

**Table 4:** Mean and SD (values in parenthesis) of amplitude and latencies of mismatch nagativity components in the auditory neuropathy group

	AN individuals			Normal subjects		
	MMN (On set)	MMN (Peak)	MMN (Off set)	MMN (On set)	MMN (Peak)	MMN (Off set)

Amplitude (in μV)	-0.068 (0.6)	-4.6 (2.1)	1.9 (3.2)	-0.071 (0.2)	-4.8 (1.5)	1.5 (2.5)
Latency (in ms)	117.3 (23.6)	186.4 (19.04)	209 (25.5)	120.5 (20.5)	180.6 (20.8)	204 (25.5)

Of the 9 subjects who had MMN, 5 of them could not behaviorally discriminate the two stimulus contrast (i.e. JND was more than 100 ms). Other 5 subjects who had no MMN also could not behaviorally discriminate the two-stimulus contrast. The MMN wave forms of those subjects who could behaviorally discriminate the stimulus contrast were virtually indistinguishable from those who did not behaviorally discriminate the contrast. This data indicate that presence of MMN does not necessarily indicate the presence of behavioral discrimination.

## Discussion

The major findings of this research were:

i) Open set speech identification scores varied considerably in individuals with AN and speech identification scores had a significant correlation with the low frequency hearing sensitivity.

ii) All subjects with AN had severe temporal processing deficits as shown by JND measurements and TMTF.

iii) In majority of AN patients cortical evoked potentials could be recorded but none of the measured evoked potential parameters had any relation with psychophysical measurements.

### Psychophysical measurements

Speech identification scores in AN individuals had good correlation with low frequency hearing sensitivity but not with the high frequency hearing sensitivity. This frequency specific correlation between hearing thresholds and speech identification scores, may be related to differential physiology between high frequency and low frequency coding. Low frequencies are usually coded by phase locked responses in type I auditory nerve fibers. Individuals with AN cannot use phase locking cues to the same extent as normal hearing listeners due to dyssynchronous firing of auditory nerve fibers. However, detection of the high frequency depends on place of excitation on basilar membrane and does not depend on the phase locking cues as much as low frequencies. We propose that, low frequency hearing sensitivity in these individuals may indicate the extent of temporal disruption in the auditory system. Its relation with speech identification scores is suggestive of importance of neural synchrony in understanding speech. This is also supported by other two observations:

i) A retrospective inspection of the data reveled, all 8 individuals who obtained speech identification scores more than 50% had their low frequency hearing sensitivity (average of 250 Hz, 500 Hz, and 1000 Hz) better than 25 dB HL and 6 individuals who had speech identification scores less than 20% had low frequency hearing sensitivity more than 40 dB HL.

ii) There was significant correlation between low frequency hearing sensitivity and peak modulation detection thresholds. Based on the above observations, we propose that low frequency hearing sensitivity in AN individuals may be the indicator of suprathreshold temporal processing deficits.

All AN individuals experienced severe difficulties in discriminating the speech stimuli that differed in time domain. As stimulus was presented at equal sensation levels to both the groups this resulted in difference in presentation levels (SPL) for each of the subjects. However, the difference in the JNDs for transition duration of syllable /da/ between two groups cannot be attributed to difference in presentation level (SPLs). It is shown that when the stimuli are sufficiently loud or at comfortable level auditory duration discrimination is independent of the intensity [14]. Individuals who had better discrimination abilities also possessed better open set speech identification scores. These findings stress the importance of perception of temporal variation in understanding speech information. Temporal processing deficits in individuals with AN are also demonstrated by poor performance on TMTF. Average peak sensitivity of individuals with AN was threefold more than the normals. Poor sensitivity to temporal modulations in these individuals is also reported by other investigators [6,8]. A significant correlation was observed between modulation detection thresholds and speech identification scores (when data from two subjects with paradoxical results were removed). This finding agrees with the results obtained from Rance et al [6], Zeng et al [7,8]

Difference between normal listeners and AN subjects in detection of modulation was more at higher modulation frequencies. The extent of temporal processing deficits were more than what is been reported for cochlear hearing loss of comparable degree [15]. This difference between two groups cannot be because of different presentation levels (SPA) used because modulation detection thresholds are reported to be stable over a wide range of intensities. In the auditory system, higher modulation frequencies are processed at auditory nerve and brainstem, whereas lower modulation frequencies are processed mainly in the thalamus and auditory cortex. As one ascends the auditory system, a neural encoding shift occurs. An emphasis on synchronous response for temporal coding exists at auditory nerve and brainstem (codes low frequencies) and less reliance on synchrony occurs as one move centrally (codes high frequencies) [16-18]. Hence, it can be expected that individuals with AN will have more problems in processing high rates of modulations which require synchronous firing of auditory nerve fibers. Inability of many the subjects to perceive amplitude modulation of 0 dB (100%) at higher modulation rates indicates the importance of temporal synchrony in auditory perception. Effects of reduced temporal fluctuations on speech perception in normal listeners have been reported previously [19]. Elevated modulation detection thresholds at slower modulation rates in combination with virtually no perception of modulations at high modulation rates are sufficient to disrupt the perception of amplitude envelop cues in normal speech. As this study measured only peak sensitivity, reduced peak sensitivity may also be due to reduced ability to perceive the amplitude changes in patients with auditory neuropathy.

### Electrophysiological measures

P_1_/N_1 _and P_2_/N_2 _complex amplitude and latency did not appear to be related to degree of hearing loss or speech identification scores. This result is in contrast to Rance et al [5] who evidenced a strong relation between presence of event related potential and speech perception scores. This difference in the results may be due to difference in subjects and the stimuli. Rance et al [5] primarily studied children younger than 92 months and were fitted with the amplification devices before 28 months of age. This may have prevented the retrograde loss of speech perception abilities. In our subjects, average age at which amplification provided was 18 years. Many of the subjects were not identified in childhood as they had near normal hearing sensitivity and were grouped as slow learners in the class. This huge gap in the auditory experience between two groups might have adversely affected the speech perception abilities of the later. Presence of LLR components with normal latency and amplitude represent the stimulus registration in the primary auditory cortex, which do not involve complex decoding and representation of the signal as it is required for the speech perception.

Large numbers of studies in last decade have established MMN as an objective electrophysiological measure of auditory discrimination (e.g. [13]). Our results of MMN and behavioral discrimination are paradoxical. Significant MMN was seen in the majority of subjects with auditory neuropathy, even though stimulus contrast could not be behaviorally discriminated. Fried et al [20] have provided evidence for the existence of preconscious perception in the visual system. Preconscious perception describes the physiological or neurological process that occurs without behavioral or conscious perception. Some evidence of preconscious perception is also reported in auditory system using MMN. Allen et al. [16] reported the presence of MMN in normal listeners for the stimulus contrast that they could not behaviorally discriminate. Presence of MMN in AN subjects who could not behaviorally discriminate the stimulus contrast supports the hypothesis that neural generators responsible for the MMN are not necessarily linked to conscious perception [21]. But all the individuals who had no MMN could not behaviorally discriminate the stimulus contrast. These two results in combination support the notion that MMN is necessary, but not a sufficient component for conscious perception of stimulus change.

Another possible explanation for the discrepancy between behavioral discrimination and MMN in some AN subjects may be related to perception of stimulus onset cues. We hypothesis that cues in the stimulus onset play a major role in the behavioral discrimination between the stimulus contrasts that differ in transition duration. Kraus et al [20] reported that perception of any change in the stimulus onset was extremely difficult in a subject with AN who had normal hearing. Hence the individuals with AN had larger JND's. We propose that MMN, which was present in some AN individuals, was elicited by the difference in the later part of the stimulus. However, it is unclear that why AN individuals could not discriminate the stimulus contrasts by using the information in the later part of the stimulus that elicited the MMN.

## Conclusion

Findings of this study indicate that individuals with AN have severely affected temporal processing abilities. These temporal processing deficits correlate significantly with the speech identification scores and hearing sensitivity in the low frequency region. Psychophysical measures including speech perception did not correlate with the electrophysiological measurements used at least in adults with AN.

## Authors' contributions

AKU was involved in designing the study, data collection, analysis, interpretation and preparing the manuscript. JM was involved in designing the study, interpretation and preparing the manuscript.
